# How relevant is lumbar bone mineral density for the stability of symphyseal implants? A biomechanical cadaver study

**DOI:** 10.1007/s00068-021-01850-6

**Published:** 2021-12-08

**Authors:** Fanny Schwaabe, Johannes Gleich, Christoph Linhart, Alexander Martin Keppler, Matthias Woiczinski, Christian Kammerlander, Axel Greiner, Wolfgang Böcker, Adrian Cavalcanti Kußmaul

**Affiliations:** 1 Department of Orthopaedics and Trauma Surgery, Musculoskeletal University Center Munich (MUM), University Hospital, LMU Munich, Marchioninistr. 15, 81377 Munich, Germany; 2 AUVA Traumahospital Styria, Graz, Austria

**Keywords:** Biomechanics, Fracture, Osteoporosis, Bone mineral density, Pull-out force, Osteosynthesis, Pubic symphysis

## Abstract

**Purpose:**

Osteoporotic bone tissue appears to be an important risk factor for implant loosening, compromising the stability of surgical implants. However, it is unclear whether lumbar measured bone mineral density (BMD) is of any predictive value for stability of surgical implants at the pubic symphysis. This study examines the fixation strength of cortical screws in human cadaver specimens with different BMDs.

**Methods:**

The lumbar BMD of ten human specimens was measured using quantitative computed tomography (qCT). A cut-off BMD was set at 120 mg Ca-Ha/mL, dividing the specimens into two groups. One cortical screw was drilled into each superior pubic ramus. The screw was withdrawn in an axial direction with a steady speed and considered failed when a force decrease was detected. Required force (N) and pull-out distance (mm) were constantly tracked.

**Results:**

The median peak force of group 1 was 231.88 N and 228.08 N in group 2. While BMD values differed significantly (*p *< 0.01), a comparison of peak forces between both groups showed no significant difference (*p *= 0.481).

**Conclusion:**

Higher lumbar BMD did not result in significantly higher pull-out forces at the symphysis. The high proportion of cortical bone near the symphyseal joint allows an increased contact of pubic screws and could explain sufficient fixation. This condition is not reflected by a compromised lumbar BMD in a qCT scan. Therefore, site-specific BMD measurement could improve individual fracture management.

**Supplementary Information:**

The online version contains supplementary material available at 10.1007/s00068-021-01850-6.

## Introduction

Anterior plating remains the preferred surgical treatment for symphyseal disruptions [1, 2]. However, complications after fixation of symphyseal injuries are frequent and wide-ranging [2]. Besides infection and patient discomfort, implant failure is the main indication for surgical revision [3].

Radiological signs of screw loosening are described in up to 81% of the patients, while therapeutical relevance remains controversial [3]. If loosening is associated with a loss of fracture reduction and symptomatic diastasis, surgical revision is indicated [4].

For comprehensive fracture management, all factors influencing stability of the applied osteosynthesis have to be considered [5–7]. Reduced bone mineral density (BMD) could affect stability of the osteosynthesis and prevails around the world due to demographic changes [8–12]. Significant risk factors for osteopenia and osteoporosis are increased life expectancy, immobilisation and previous fractures [13]. BMD is an important parameter in diagnostics of osteopenia and osteoporosis, allowing the evaluation of fracture risk in the elderly and enabling monitoring of an existing osteoporosis therapy [13, 14]. It is commonly assessed via dual-energy X-ray absorptiometry (DXA) or quantitative computed tomography (qCT) at the lumbar spine [15, 16]. Biomechanical studies presented that some implants are more likely to malfunction in models with lower bone density than in those with higher bone density [7]. Seebeck et al. concluded that cortical thickness and cancellous density have a significant effect on the retention force of screws for axial pull-out loading on human tibiae [6]. Other studies demonstrated a significant positive correlation of BMD with the insertion torques of cortical bone screws in human cadaveric femoral bones and of trabecular BMD with pull-out forces at the proximal humerus [17, 18]. In contrast, Choe et al. did not find a significant correlation between lumbar measured t-scores and different regions of the femur in osteoporotic patients [11]. Similarly, Cummings et al. showed that bone density measured at the distal radius or the spine is not as accurate for assessing the risk of hip fracture than values measured at the femoral neck itself [19].

This study aims to evaluate the influence of decreased lumbar BMD, assessed via qCT, its consecutive transferability and potential predictive value for the stability of symphyseal cortical screws.

The authors hypothesize that lumbar BMD, determined via qCT, has no significant influence on the pull-out strength of cortical screws used in symphyseal plating.

## Materials and methods

Ten fresh-frozen human cadaveric anterior pelvic rings were used in this biomechanical study. Approval of the local ethics committee (approval no. 210-16) as well as of the donors' relatives was obtained prior to the investigation. The specimens included one female and nine male donors ranging from 25 to 74 years with a mean age of 60 years.

First, the bone mineral density of each pelvis was determined at the lumbar vertebrae 4 and 5 using a qCT. The mean BMD was 120.27 mg Ca-Ha/mL (range 63.2–171.0 mg Ca-Ha/mL, median 123.70 mg Ca-Ha/mL). The specific characteristics of the specimens are provided in Table 1.

**Table 1:** Characteristics of the specimens including age, sex and the average BMD of the pelvis specimens as determined by quantitative computed tomography (qCT)

Specimen	Age (years)	Sex	Average BMD (mg Ca-Ha/mL)	Pull-out force (N)
1	65	M	64.9	199.59
2	51	F	171.0	78.8
3	60	M	121.6	133.18
4	57	M	152.5	226.18
5	25	M	151.7	632.8
6	74	M	104.6	190.56
7	67	M	63.2	264.18
8	72	M	113.7	300.36
9	64	M	125.8	229.99
10	65	M	133.7	579.40

**Fig. 1:**
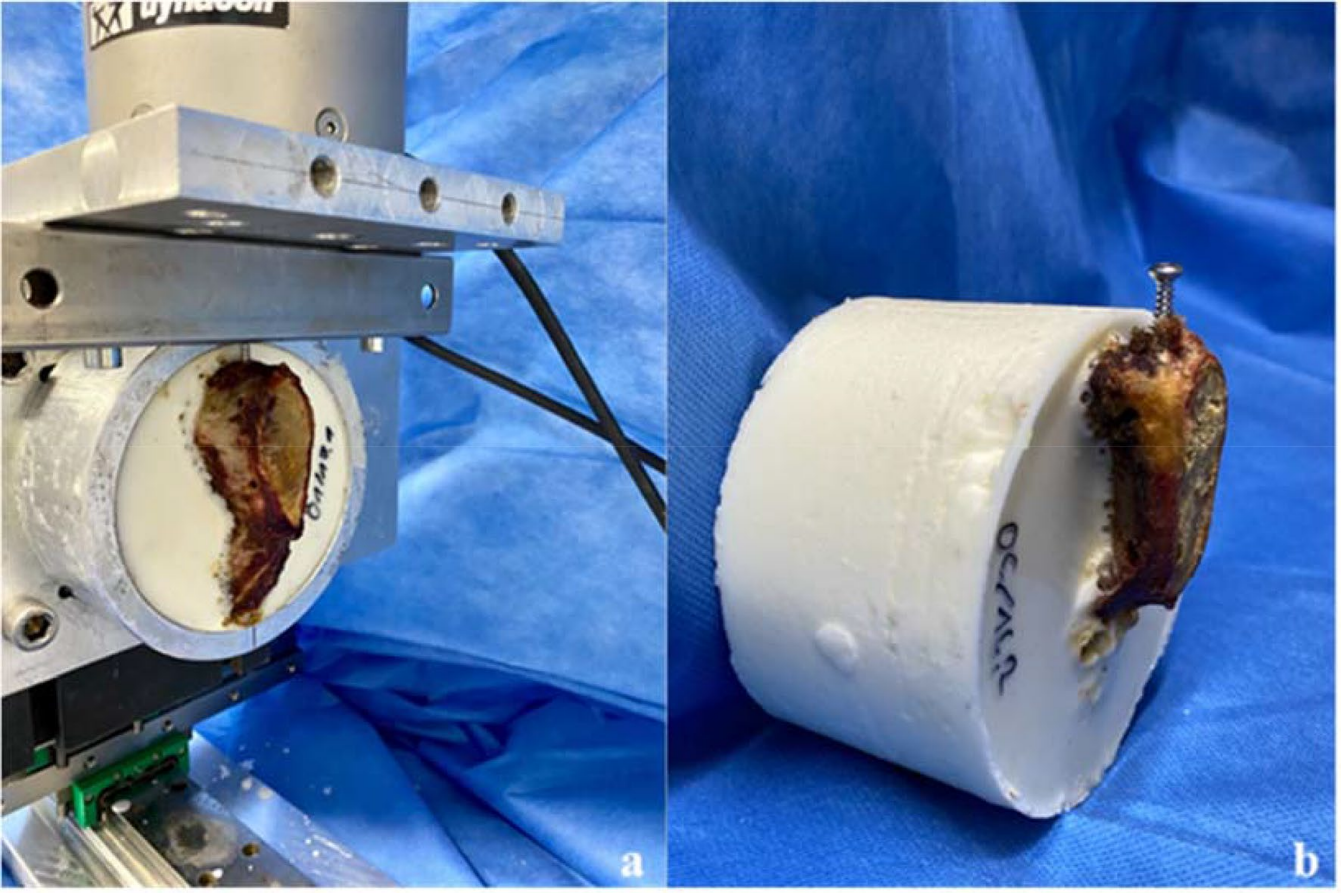
Specimen embedded and mounted on the Instron testing machine during testing (**a**) and after screw pull-out (**b**)

After thawing the pelvises one day in advance, they were heated in a water bath at 35 °C for 30 min immediately prior to testing to mimic body temperature. The anterior pelvic rings were then embedded in a metal cylinder filled with epoxy resin and mounted on an Instron testing machine (Instron ElectroPulsTM E10000 Linear-Torsion, Norwood, MA, USA) (Fig. 1). To ensure an exact vertical orientation of the pubic symphysis, a symphyseal plate (DePuy Synthes 3.5; four holes, dynamic compression plate, Raynham, Massachusetts, USA) was placed onto the symphyseal joint throughout the embedding and the prospective screw entry point of the medial parasymphyseal screw was marked.

**Fig. 2:**
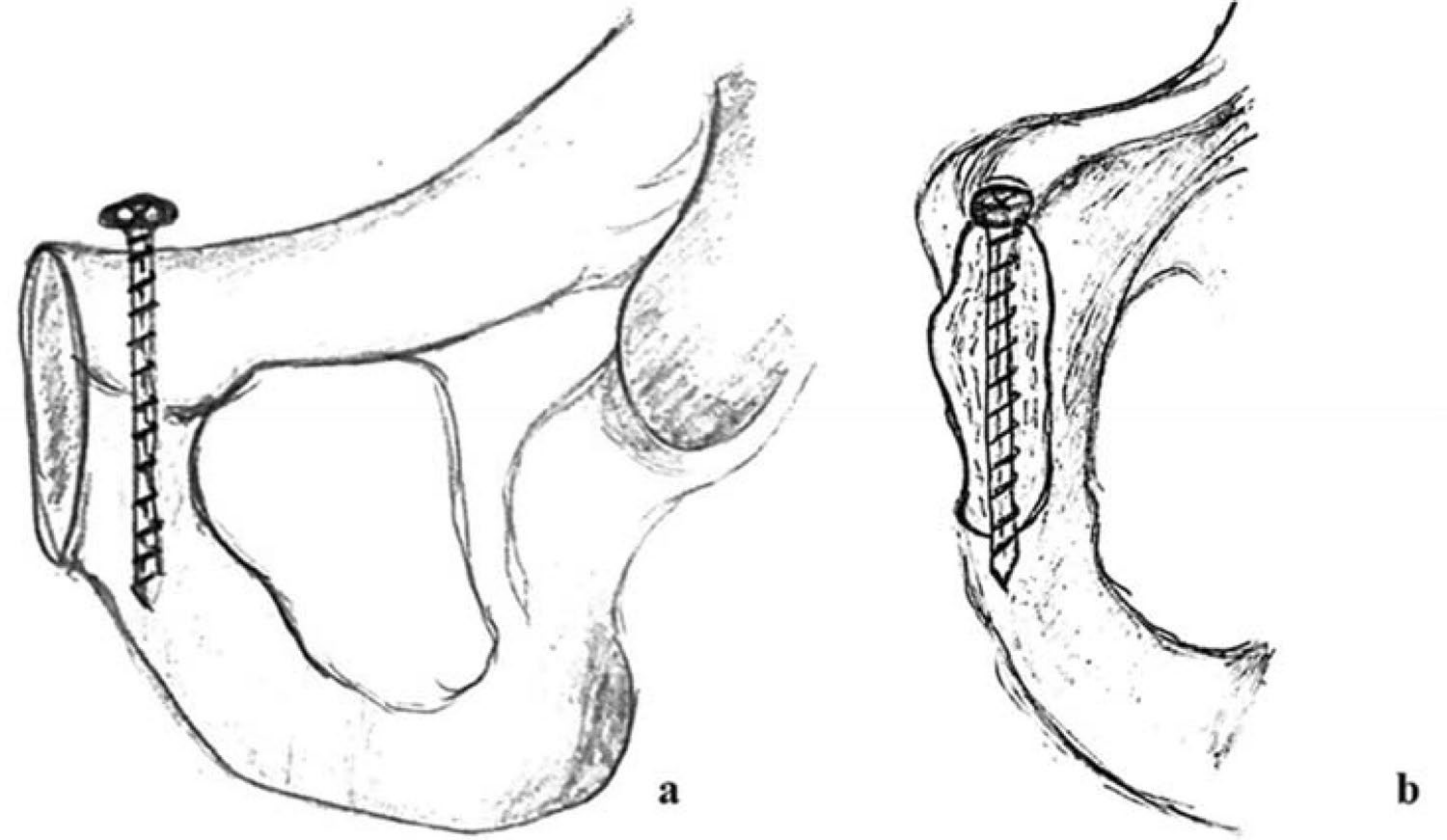
Draft of the cortical screw position from frontal (**a**) and medial (**b**)

Next, a cortical screw (DePuy Synthes Cortex Screw 3.5 mm, 50 mm) was accordingly drilled in a 90-degree angle to the former plate position into the superior pubic ramus parallel to the symphyseal joint (Fig. 2). This way, an exact vertical pull-out for each screw was ensured. Each screw was placed monocortically 40 mm into the bone using a 10 mm spacer.

**Fig. 3:**
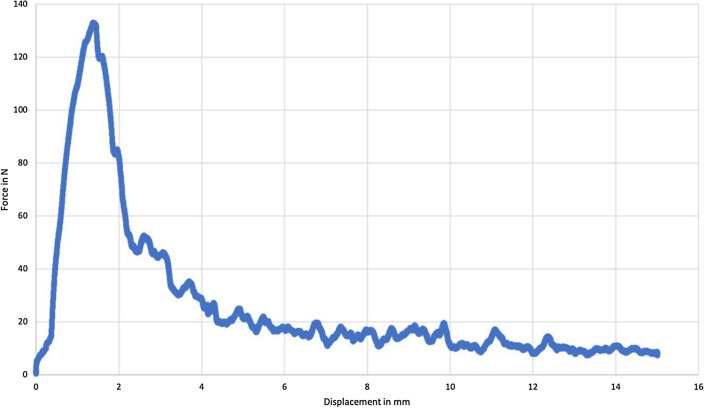
Exemplary force–displacement curve (specimen 3)

To reduce traction forces occurring when attaching the testing machine to the screw and potentially confounding the results, a tension of 7 ± 3 N was applied to every screw before starting the test protocol. The screw was then pulled out in an axial direction with a steady speed of 10 mm/min based on a test protocol by Jöckel et al. and was considered failed when detecting a decrease in axial pull-out force and a concomitant loss of screw resistance (Fig. 3) [20]. The required force (N) during the extraction and the dislocation distance (mm) of the screw were permanently tracked. Similar to other studies, the pull-out strength was defined as the peak value of the force–displacement curve [6, 21] (Fig. 3)*.*

For better data comparison, a cut-off value for bone density was set at 120 mg Ca-Ha/mL, dividing the specimens into two groups. Accordingly, specimens with a density below this cut-off formed group 1 (*n* = 4), while those with higher values formed group 2 (*n* = 6). The cut-off at 120 mg Ca-Ha/mL for bone density was chosen arbitrarily to ensure nearly equal sample sizes in each group. Subsequently, peak force values from both groups were compared.

Statistical analysis was performed with IBM SPSS Statistics (Windows, version 26.0, IMB Corp., Armonk, N.Y., USA). Determination of normal distribution was performed using the Kolmogorov–Smirnov, Shapiro–Wilk test as well as graphically, if needed. In case of normal distribution, homogeneity of variance was verified using Levene’s test. In case of homogeneity of variance, a *t* test was used. If variables showed heterogeneity of variance, a Welch test was carried out for comparison. Pearson correlation coefficient was used to evaluate linear correlation both between BMD and pull-out force and between BMD and age. Two tailed *p *< 0.05 was considered as significant.

## Results

**Fig. 4:**
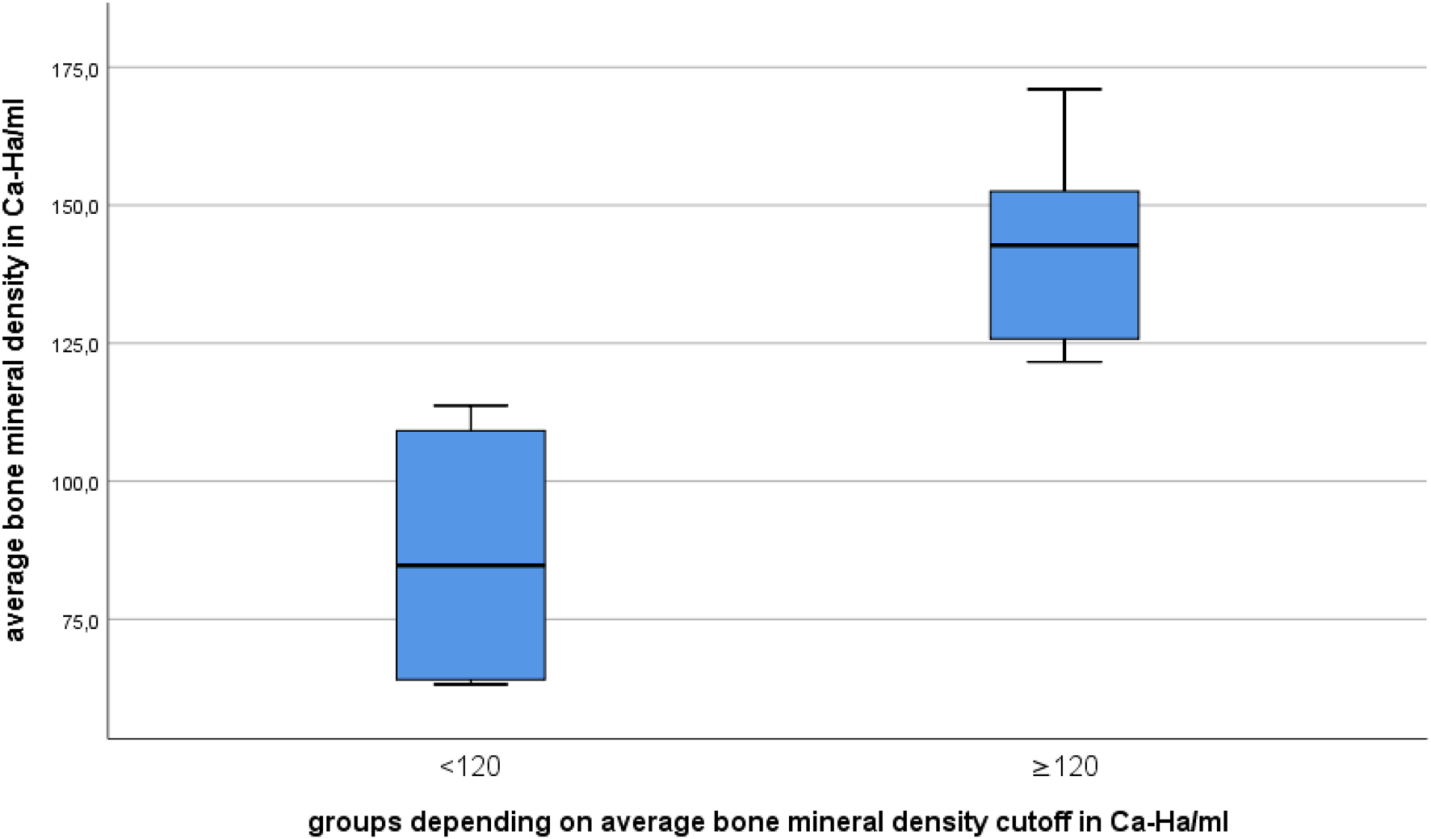
Comparison of the average bone density between group 1 and 2 with respect to cut off value

Group 1 (BMD < 120 mg Ca-Ha/mL) showed an average BMD of 86.60 ± 26.31 mg/mL, while group 2 (BMD ≥ 120 mg Ca-Ha/mL) displayed an average BMD of 142.72 ± 18.92 mg Ca-Ha/mL (*p *< 0.01) (Fig. 4). According to reference values in the dual-energy computed tomography (DECT) examination, bone density < 70 mg HA/cm^3^ can be interpreted as osteoporosis, values from 70 to 110 mg HA/cm^3^ as osteopenia and values > 110 mg HA/cm^3^ indicate normal bone density [22]. Following this definition, two specimens showed osteoporosis (Nr. 1,7) and one osteopenia (Nr. 6).

**Fig. 5:**
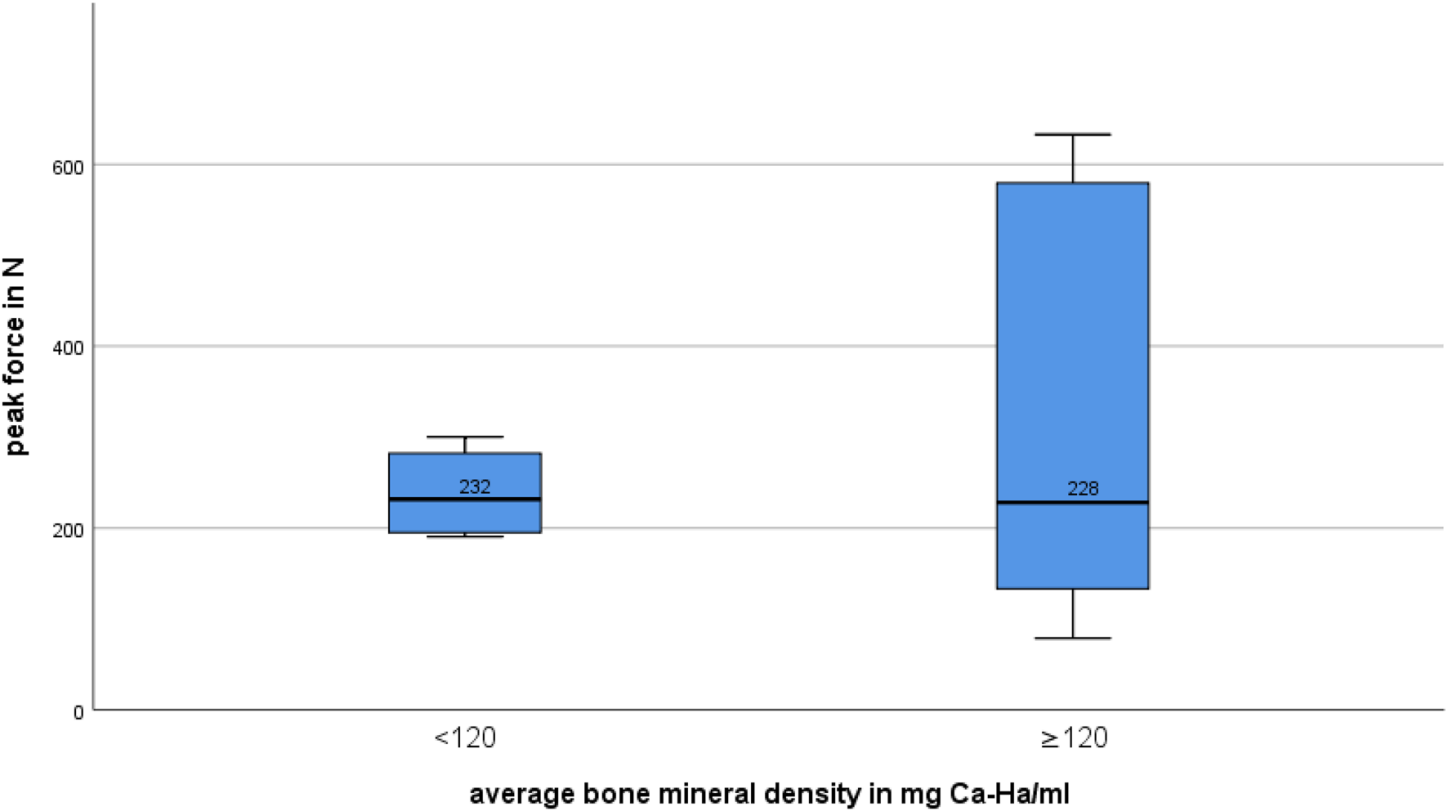
Boxplot of the peak force values

The average pull-out peak force of group 1 was 238.67 ± 52.60 N, whereas group 2 had a mean pull-out peak force of 313.39 ± 234.46 N (*p *= 0.481) (Tables 1, 2). The median peak force of group 1 was 231.88 N and 228.08 N in group 2 (Fig. 5).

**Table 2:** Peak forces and average bone mineral density; Group 1 (BMD < 120 mg Ca-Ha/mL); Group 2 (BMD ≥ 120 mg Ca-Ha/mL)

	Group 1	Group 2
Average BMD in mg Ca-Ha/mL	86.60 ± 26.31	142.71 ± 18.92
Average force peak in N	238.67 ± 52.6	313.39 ± 234.46
Median force in N	231.88	228.08

Considering the nine specimens older than 51 years while excluding the youngest one (25 years), average bone mineral density and age showed an inverse correlation (Pearson correlation coefficient *r* =  − 0.641). Bone mineral density and pull-out force showed a positive correlation (*r* = 0.160).

## Discussion

The prevalence of screw loosening after anterior fixation of the pubic symphysis is high and may lead to implant failure. Therefore, possible reasons should be analysed to prevent loosening, increase stability of osteosynthesis and improve patient’s outcome.

Osteoporosis compromises stability of implants at different sites (well-known at the spine or the proximal femur); thus the pubic symphysis might also be affected [8]. Osteoporosis is defined by a loss of bone mass and destruction of its microarchitecture and is associated with increased fracture risk [5, 9, 23]. To assess individual fracture risk, different ways measuring bone mineral density are available, such as a qCT scan at the lumbar spine [15, 16]. If any correlation of BMD and pull-out strength of screws at the pubic bone could be observed, patients at risk for implant failure could be identified in advance.

In this biomechanical study no significant correlation between lumbar BMD and pull-out force and no significant difference in pull-out forces between two groups of specimens with different BMD could be observed (Fig. 4). Arbitrary categorization of tested specimens, using a BMD cut-off at 120 mg Ca-Ha/mL, resulted in two groups with a significant difference in mean BMD (*p *< 0.01) (Fig. 4). Increased bone mineral density did not result in increased pull-out forces at pubic symphysis.

Several reasons for the restricted predictive value of lumbar determined BMD for fixation strength of screws in symphyseal osteosynthesis are possible as follows:

First, distribution of bony structures is different in vertebral and pelvic bone: while vertebral bodies mostly consist of trabecular bone, pubic bone is primarily composed of compacta [24]. This leads to increased contact surface of the pubic screw with cortical, and consequently more substantial bony structures, which may explain a higher resistance during pull-out, independent of lumbar bone quality. Furthermore, age of onset and severity of bone density loss between trabecular and cortical bone vary widely [24]. This different ageing behaviour implies that bone loss, which is already present in the spine, may not have reached the same extent in the appendicular skeleton [24]. This might explain that both groups showed almost equal median pull-out forces, even though there was a significant difference in BMD values.

Several studies have already underlined limited transferability of bone density values measured at different sites of the skeleton and stated to assess BMD at the site of interest: Choe et al. observed no significant correlation between T-scores of lumbar vertebrae and different regions of the femur in female osteoporotic patients [11]. Other groups found a positive correlation between trabecular bone mineral density of the humeral head and pull-out strength at the same site; also BMD measured at the femoral neck was more accurate for assessing the risk of hip fracture than density values gained at the radius or spine and in-situ DXA at the same skeletal spot was more likely to be related to fracture loads than at remote sites [18, 19, 24]. Bredow et al. indicated a potential benefit of preoperative bone density determination using a CT scan as a decisional aid for possible extension of surgical techniques (e.g. cement augmentation) to prevent screw loosening [25]. Also, preoperative assessment of local cancellous bone density has been described as feasible aid in the treatment of proximal femur fractures [26].

Second, bone density loss due to osteoporosis does not affect the whole organism equally [27]. Predisposing sites include bones rich of spongiosa, like the axial skeleton (vertebral bodies), the femoral neck or distal radius [23, 28]. This assumption is strengthened by a recent study, concluding that a decrease in BMD due to osteoporosis differs in time and severity at various sites of the skeleton [29]. In the present study, a decrease of average BMD with increasing donors age was observed, underlining the representativity of the specimens [29, 30]. Excluding specimen Nr. 5, there was a significant inverse correlation, which indicates a large effect size [31].

Finally, and with regard to the clinical relevance of this study, it is essential to keep in mind that compromised bone quality is only one risk factor for implant failure: symphyseal plating almost completely eliminates the physiological range of motion of the symphyseal joint (up to 2 mm), resulting in an iatrogenic arthrodesis and consequently increasing the rate of implant failure [32]. Therefore, further options to improve the stability of screws for the treatment of symphyseal injuries should be explored. For example, an additional cement augmentation might increase fixation strength and reduce implant failure. This concept has already been successfully implemented in a study by Suero et al. at the posterior pelvic ring: here, a single augmented screw achieved comparable stability to a non-augmented double-screw technique [33]. Weiser et al. demonstrated a significant increase in fatigue strength of cement augmented screws in osteoporotic vertebrae [34, 35].

Another, potentially biomechanically superior, option could be the insertion of minimally invasive Tape-Suture constructs: these constructs have demonstrated sufficient biomechanical stability while allowing the above-mentioned micromovements of the pubic symphysis as shown by previous studies of our group [36].

Overall, this study demonstrates the importance of site-specific densitometry regarding the corresponding implant stability and highlights the relevance of the inclusion of diverse manifestations of osteoporosis and osteopenia in the therapeutical concept.

The main limitation of this study is a relatively small sample size and an underrepresentation of osteopenic and osteoporotic BMD. Also, there is an uneven representation of male and female specimens as well as ethnicities. The opportunity to test human cadaveric specimens, however, is a great privilege and entails a very high moral obligation towards the donors. It would be unethical to reject samples to achieve an ideal ratio of osteoporotic and non-osteoporotic bone or other characteristics. The cut off value to create approximately equal sample sizes and enable better comparison was set arbitrarily and not after defined criteria, which is reasonable regarding the ethical standards mentioned above.

Another limitation was the use of identical screw lengths in all pelvises (DePuy Synthes Cortex Screw 3.5 mm, 50 mm) and the monocortical screw positioning. In a clinical setting, an individual screw length would be measured prior to insertion and a bicortical fixation attempted. This approach would have resulted in decreased comparability as the individual variation in screw length necessary to achieve bicortical screw positioning would have led to a variation in fixation strength due to the differences in thread pitches.

Yet, potential confounding variables were discussed with the biomechanical engineers prior to this study: consequently, monortical and axial screw alignment of a single symphyseal screw, identical screw length and vertical screw pull-out were chosen to minimize confounding variables, to achieve standardization of the experimental set-up and to consequently maximize comparability of the data.

## Conclusion

In this biomechanical study, we were able to demonstrate that bone mineral density measured at the lumbar spine with a qCT does not allow sufficient conclusion about the fixation strength of cortical screws at the pubic symphysis. Site-specific performed densitometry should be evaluated in further studies to potentially draw conclusions for subsequent implant stability.

Also, the authors underline the importance of the inclusion of profound knowledge about the diverse manifestations of osteoporosis and osteopenia into the surgical concept.

## Supplementary Information

Below is the link to the electronic supplementary material.

Supplementary file1 (XLSX 16332 KB)

## Data Availability

All data is available upon request.
